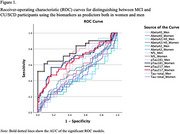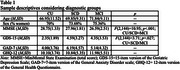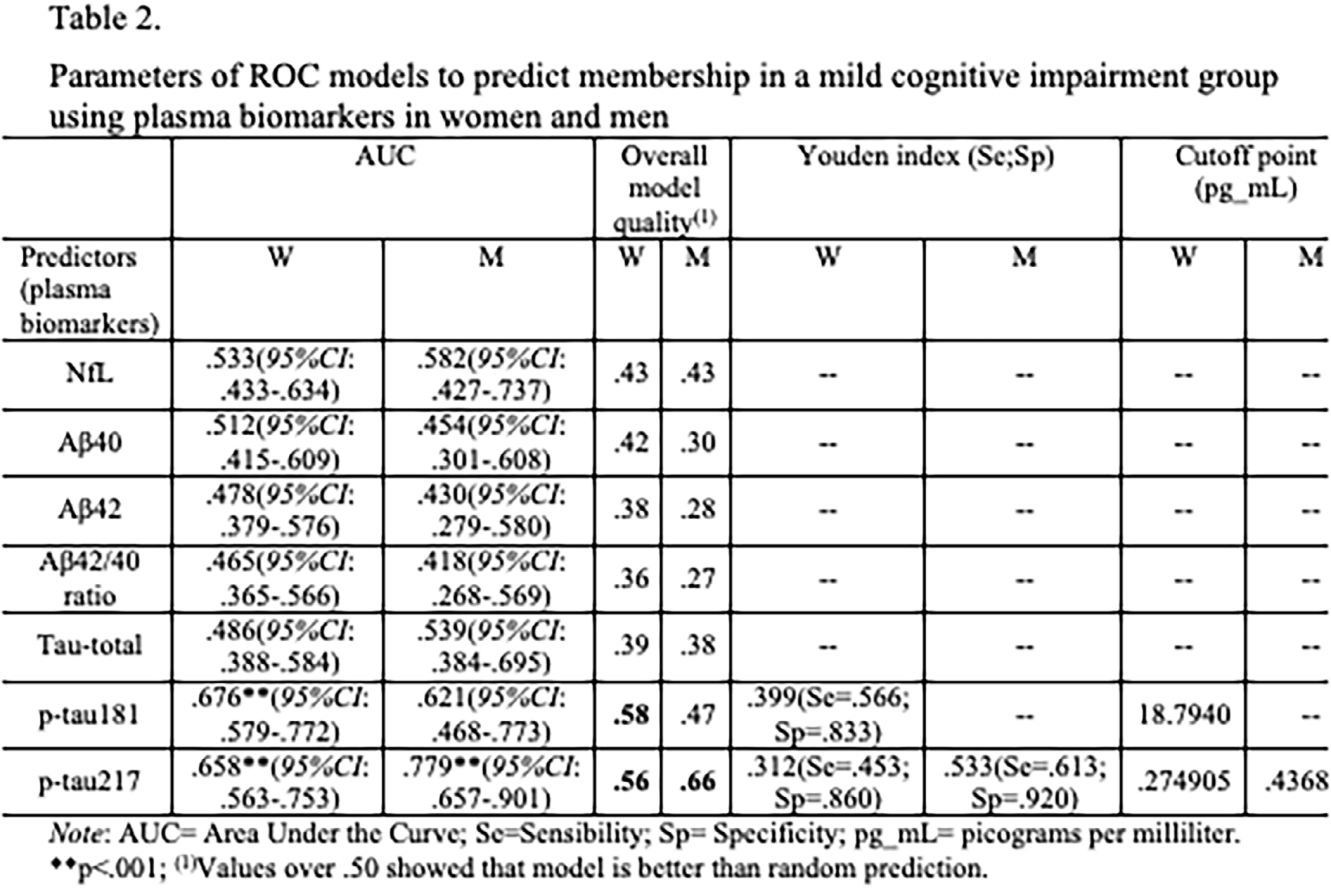# Plasma Aβ40, Aβ42, Aβ42/40 ratio, tau, *p*‐tau181, *p*‐tau217 and NfL biomarkers to discriminate the prodromal stage of dementia along the continuum of cognitive impairment: Sex differences in the CompAS study

**DOI:** 10.1002/alz70856_104759

**Published:** 2026-01-07

**Authors:** Ana I. Rodríguez‐Pérez, Juan J. Ansede‐Bermejo, Cristina Lojo‐Seoane, Maria Campos‐Magdaleno, Ana Nieto‐Vieites, Alba Felpete, Santiago Galdo‐Álvarez, Mónica Lindín, Águeda Rojo‐Pantoja, M. José Moreno‐Carretero, Jose M. Aldrey, Carlos Spuch, Juan M. Pías‐Peleteiro, Angel Carracedo, Jose Luis Labandeira, Montserrat Zurrón, David Facal, Fernando Díaz, Arturo X. Pereiro Rozas

**Affiliations:** ^1^ Centro Singular de Investigación en Medicina Molecular e Enfermidades Cronicas (CIMUS), Universidade de Santiago de Compostela, Santiago de Compostela, Galicia, Spain; ^2^ Instituto de Investigación Sanitaria de Santiago de Compostela (IDIS), Santiago de Compostela, Galicia, Spain; ^3^ Centro de investigación Biomédica en Red sobre Enfermedades Neurodegenrativas (CIBERNED), Santiago de Compostela, Gallicia, Spain; ^4^ Centro Nacional de Genotipado (CEGEN‐PRB3‐ISCIII), Universidade de Santiago de Compostela, Santiago de Compostela, Galicia, Spain; ^5^ Fundación Pública Galega de Medicina Xenómica‐ CIBERER‐IDIS, Santiago de Compostela, Spain; ^6^ Instituto de Psicoloxía (IPsiUS), Universidade de Santiago de Compostela, Santiago de Compostela, Galicia, Spain; ^7^ Departamento de Psicoloxía Evolutiva e da Educación, Universidade de Santiago de Compostela, Santiago de Compostela, Galicia, Spain; ^8^ Applied Cognitive Neuroscience and Psychogerontology group, Health Research Institute of Santiago de Compostela (IDIS), Santiago de Compostela, Spain; ^9^ Instituto de Psicoloxía (IPsiUS), Universidade de Santiago de Compostela, Santiago de Compostela, Spain; ^10^ Universidade de Santiago de Compostela, Santiago de Compostela, Spain; ^11^ Galicia Sur Health Research Institute (IISGS), Vigo, Pontevedra, Spain; ^12^ Health Research Institute of Santiago (IDIS), Santiago de Compostela, Spain; ^13^ Galicia Sur Health Research Institute (IISGS), Vigo, Spain; ^14^ Research Health Institute of Santiago (IDIS), Santiago de Compostela, Spain; ^15^ Grupo de Medicina Xenómica, Centro Nacional de Genotipado (CEGEN‐PRB3‐ISCIII). Universidade de Santiago de Compostela, Santiago de Compostela, Spain; ^16^ Research Center for Molecular Medicine and Chronic Diseases (CIMUS), University of Santiago de Compostela, Santiago de Compostela, Spain; ^17^ Networking Research Center on Neurodegenerative Diseases (CIBERNED), Santiago de Compostela, Spain

## Abstract

**Background:**

Plasma Aβ40, Aβ42, Aβ42/40 ratio, tau, *p*‐tau181, *p*‐tau217, and NfL have been proposed as possible valid early blood biomarkers for Alzheimer's Dementia (AD) (Simren et al., 2021). Although sex differences in some biomarkers (e.g., *p*‐Tau‐181) were suggested in some studies (Tsiknia et al., 2022), this issue remains largely unexplored. Our aim was cross‐sectionally analyze predictive associations of the biomarkers to differentiate MCI from preclinical stages (CU, SCD) of the AD continuum in both women and men.

**Method:**

Sample consisted of 145 participants (CU=20; SCD=57; MCI=68), mostly women (73.7%) and with age media above 70 years (*M* = 70.39; *SD* = 9.62) (see Table 1 for sample descriptives).

Peripheral blood samples drawn into EDTA tubes, centrifuged, and plasma samples aliquoted and kept at –80◦C according to standardized biobanking protocols.

Approximately seven months after storage, the concentrations of NfL, Aβ‐40, Aβ‐42, tau, *p*‐tau217, and *p*‐tau181 were quantified using the ultra‐sensitive Single Molecule Array (SIMOA) technology on the Simoa SR‐X platform (Quanterix). The corresponding commercial kits (PCs. 104073, 101995, 104570, and 104111) were used in strict accordance with the manufacturer's protocol.

Receiver operating characteristics (ROC) for women and men were plotted to test how well the risk prediction model discriminates between participants with MCI and CU/SCD using the considered biomarkers.

**Results:**

ROC curves showed significant models only for *p*‐tau181 and *p*‐tau217 biomarkers in discriminate MCI participants and only *p*‐tau217 was valid for women and men. Modest AUCs were obtained, ranging between .62 and .79, for these significant models. Our results only showed significant predictive association between MCI and *p*‐tau181 for women. A better classification of men with MCI was achieved using the biomarker *p*‐tau217 (Se=.613; Sp=.920), while for women better accuracy was achieved with *p*‐ Tau‐181 (Se=.566; Sp = .833), although specificity was better than sensitivity in both models.

**Conclusions:**

Our results supported the existence of sex differences in predictive association of *p*‐tau181 and differences in the validity of *p*‐tau217 to discriminate women and men with MCI. Sex differences in plasma concentrations of *p*‐tau‐217 and, mainly, *p*‐tau181 may confound the interpretation of these biomarkers and reduce the validity of diagnoses when sex is not considered.